# Pregnancy-Related Cardiovascular Diseases: A Radiological Overview

**DOI:** 10.3390/jcdd12020043

**Published:** 2025-01-25

**Authors:** Francesco Lauriero, Giulia Mazza, Alessio Perazzolo, Giacomo Ottoni, Alessia Cipriani, José F. Castro Pereira, Riccardo Marano, Luigi Natale

**Affiliations:** 1 Department of Diagnostic Imaging, Oncological Radiotherapy and Hematology, Fondazione Policlinico Universitario Agostino Gemelli IRCCS, Largo Agostino Gemelli 8, 00168 Rome, Italy; riccardo.marano@policlinicogemelli.it (R.M.); luigi.natale@policlinicogemelli.it (L.N.); 2 Department of Radiological and Haematological Sciences-Section of Radiology, Università Cattolica del Sacro Cuore, 00168 Rome, Italy; giulia.mazza05@icatt.it (G.M.); alessio.perazzolo01@icatt.it (A.P.); giacomo.ottoni01@icatt.it (G.O.); alessia.cipriani03@icatt.it (A.C.); 3 Department of Radiology, Unidade Local de Saúde de Almada-Seixal, E.P.E., 2805-267 Almada, Portugal

**Keywords:** pregnancy, cardiovascular diseases, pulmonary embolism, aortic dissection, spontaneous coronary artery dissection, peripartum cardiomyopathy, magnetic resonance imaging (MRI), computed tomography (CT)

## Abstract

Pregnancy induces significant hemodynamic changes, and cardiovascular diseases (CVDs) are one of the leading causes of non-obstetric maternal morbidity and mortality during pregnancy or the postpartum period in developed countries. The effective diagnosis and management of CVDs in pregnant women require a thorough evaluation that considers the health of both the mother and the fetus. Imaging plays a pivotal role in this evaluation, offering essential insights into the most significant pregnancy-related CVDs. However, due to concerns about fetal exposure, the use of contrast agents and radiation exposure must be carefully managed. Following to the principle of “As Low As Reasonably Achievable” (ALARA), strategies to minimize these risks are crucial for ensuring patient safety while maintaining diagnostic accuracy. This review highlights the contribution of cardiovascular imaging techniques, particularly computed tomography (CT) and magnetic resonance imaging (MRI), in the assessment of common pregnancy-related CVDs, and outlines strategies to reduce radiation exposure and limit contrast agent use when feasible, aiming to increase radiologists’ awareness of this crucial topic.

## 1. Introduction

The cardiovascular system undergoes predictable hemodynamic and structural changes during pregnancy, including increased plasma volume, higher cardiac output, and reduced maternal systemic vascular resistance [1]. Pregnancy is also a hypercoagulable state, resulting from increased levels of procoagulant factors along with lower anticoagulant activity [2]. These changes are especially impactful in women with pre-existing cardiovascular diseases (CVDs), who should receive counseling on maternal and fetal risks prior to conception. The modified World Health Organization (mWHO) classification should be used to assess the maternal risk of cardiovascular complications during pregnancy. Women are classified into four categories based on specific congenital and acquired CVDs, with mWHO Class IV representing an extremely high risk of maternal mortality or severe morbidity, for which pregnancy is contraindicated [3]. CVDs are the leading cause of indirect maternal mortality [4]. A careful clinical assessment is essential for the effective management of CVDs in pregnant women, with imaging playing a key role in risk stratification, diagnosis, and monitoring. The choice of imaging techniques should prioritize diagnostic accuracy while ensuring the safety of both the mother and fetus. Echocardiography is the preferred imaging method during pregnancy and the most widely used, followed by magnetic resonance imaging (MRI). In selected cases, chest radiography, computed tomography (CT), and invasive coronary angiography (ICA) may also be necessary. The primary concern with imaging in pregnancy is the exposure to ionizing radiation and the use of contrast agents (CAs) [5]. When advising pregnant patients requiring diagnostic imaging, radiologists should provide a clear explanation of radiation exposure, discuss the potential benefits and risks associated with various imaging modalities, and address any further questions or concerns related to the recommended procedures. The aim of this review is to explore the most common pregnancy-related CVDs that radiologists may encounter in routine practice. We emphasize the role of CT and MRI in diagnosis and management, particularly focusing on the administration of CAs during pregnancy and lactation. Additionally, we outline strategies to minimize radiation exposure and limit CAs’ use whenever feasible, in order to enhance radiologists’ awareness of this critical topic.

## 2. Contrast Agents: Safety Aspects

When contrast agent administration is required for diagnostic purposes in pregnant or lactating woman, radiologists must be thoroughly aware of the available data regarding the benefits and risks of the procedure. Close collaboration with the referring physicians, who order the examination, is essential to ensure the health and safety of both the mother and fetus.

### 2.1. Iodine-Based Contrast Agents

Iodinated contrast media have been shown to cross the human placenta and enter the fetus in measurable quantities [6]. In vivo studies in animals have reported no teratogenic or mutagenic effects from the use of iodinated contrast media in pregnancy, and there is no evidence suggesting that these agents are teratogenic in humans [7]. Most iodinated CAs are classified as pregnancy category B drugs by the Food and Drug Administration (FDA), with the exception of diatrizoate meglumine and diatrizoate meglumine sodium, the parenteral forms of which are listed as category C [8]. The use of nonionic contrast media in pregnant women does not appear to impact neonatal thyroid-stimulating hormone (TSH) levels, likely because the excess iodide in the fetal circulation is minimal and transient [9]. No cases of neonatal hypothyroidism have been documented as a result of maternal intravascular injection of water-soluble iodinated CAs [10]. According to the 2018 European Society of Urogenital Radiology (ESUR) guidelines on contrast agents, iodine-based contrast media may be administered to pregnant women when necessary for diagnostic purposes and should be limited to situations in which the diagnostic information obtained will alter care. Since routine screening for congenital hypothyroidism is performed on all newborns within the first week of life, it is recommended that those exposed to contrast media during pregnancy be closely monitored for this condition [11]. Similar recommendations are provided in the latest American College of Radiology (ACR) manual on contrast media [7]. For lactating women receiving iodine-based CAs, available data suggest that it is safe for the mother and infant to continue breastfeeding, as the percentage of iodinated contrast medium excreted into the breast milk and absorbed by the infant’s gut is very low (under 0.01%) [12]. Therefore, when CAs are administered to a lactating mother, breastfeeding may continue normally [7,11]. However, the decision to temporarily stop breastfeeding should be left to the mother, as survey data indicate that patient preferences and radiologists’ opinions may sometimes differ [13]. If the mother remains concerned about any potential adverse effects on the infant after being properly informed, the consensus recommendation is that she should pump breast milk prior to CA administration and discard the milk produced in the 12 to 24 h following the procedure [7].

### 2.2. Gadolinium-Based Contrast Agents

Gadolinium-based contrast agents (GBCAs) can be classified into two categories based on their chemical structure: linear and macrocyclic. Macrocyclic agents generally have lower dissociation constants and exhibit less retention in the body compared to linear agents [14]. Although gadolinium is administered in a chelated form to prevent toxicity, high or repeated doses in animal studies have shown teratogenic effects, likely due to gadolinium dissociating from the chelate [15]. Gadolinium is water-soluble and can cross the placenta into the fetal circulation and amniotic fluid, as demonstrated by studies in animals [16]. Once in fetal blood circulation, gadolinium is filtered by the fetal kidneys and excreted into the amniotic fluid through urination. This process partially allows gadolinium to enter maternal circulation, and when the fetus swallows the amniotic fluid, it can re-enter the fetal digestive system and circulation [17]. No known mutagenic or teratogenic adverse effects have been reported in human fetuses following the administration of clinically recommended doses of GBCAs to pregnant women [18]. However, Ray et al. noted an increased risk of stillbirth and neonatal death, as well as a higher likelihood of rheumatologic, inflammatory, or infiltrative skin conditions in offspring following GBCA exposure during pregnancy, indicating that further studies are needed [19]. The FDA classifies gadolinium as a class C drug [8]. Consequently, the current standard of practice is to avoid routine administration of GBCAs during pregnancy due to the unknown risks of fetal exposure. The decision to administer GBCAs to a pregnant woman should be made only when there is potential for significant clinical benefit that outweighs the unknown risks of fetal exposure; this decision must follow a thorough discussion with both the referring provider and the patient [7,15,20]. For lactating women receiving GBCAs, current guidelines suggest that breastfeeding does not need to be interrupted after gadolinium administration [7]. Less than 0.04% of the administered GBCA appears in breast milk within 24 h due to its water solubility. Of this small amount, infants absorb less than 1% through their gastrointestinal tract, resulting in an expected systemic dose absorbed by the infant from breast milk of less than 0.0004% of the intravascular dose given to the mother [21]. However, some European guidelines allow for the possibility of a 24 h interruption of breastfeeding following a dose, in consultation with the physicians. In this case, the mother should be advised to express and discard breast milk from both breasts after contrast agent administration until breastfeeding resumes [11].

## 3. Pulmonary Embolism (PE)

Acute PE remains one of the leading causes of maternal death in high-income countries. According to a WHO systematic analysis, approximately 14% of all maternal deaths in developed regions are attributed to embolism [22]. The incidence of PE in pregnancy is about five times higher than in non-pregnant women of the same age [23]. Physiological changes during pregnancy and the postpartum period—including hypercoagulability, venous stasis, and vascular damage—contribute to an increased risk of PE [24]. Clinical assessment of suspected PE during pregnancy can be challenging as symptoms, like shortness of breath or tachycardia, may overlap with those of normal pregnancy. Additionally, the biomarker D-dimer has limited utility, with levels continuously increasing during pregnancy as gestation progresses [25]. Established algorithms to estimate the pretest probability for PE in the general population, such as the Wells score, have historically not been validated in pregnant or postpartum populations [26]. To better estimate the pretest probability of PE in pregnant women, recent studies have introduced efficient tools such as the pregnancy-adapted Geneva (PAG) score [27]. Current European Society of Cardiology (ESC) guidelines provide a dedicated diagnostic workup for suspected acute PE in women who are pregnant or ≤six weeks postpartum [28]. We discuss the specific role and characteristics of Computed Tomography Pulmonary Angiography (CTPA). Furthermore, we acknowledge the emerging role of non-contrast MRI in detecting PE, as its diagnostic potential is increasingly gaining attention.

### 3.1. CTPA Diagnostic Value

In the Cochrane review, the median negative predictive value for CTPA in pregnant women was 100%, and the median sensitivity was 83% [29]. This very high negative predictive value must be interpreted in the context of the low prevalence of PE in the studies evaluated (1–7%), implying a very low post-test probability of PE [30]. Further studies have confirmed that CTPA is suitable for ruling out PE during pregnancy [31]. However, inconclusive studies pose a challenge, with approximately 4% to 33% of investigations resulting in non-diagnostic findings [29,31]. The hemodynamic and structural changes a woman undergoes during pregnancy, such as increased cardiac output and diaphragm elevation due to progressive uterine distension, can lead to methodological limitations, including poor pulmonary arterial opacification and artifacts from respiratory motion [32]. Therefore, optimizing the imaging protocol is essential to improve image quality and increase diagnostic yield. This optimization includes using a higher flow rate (6 mL/s instead of 4 mL/s), increasing the contrast volume by approximately 25% followed by a saline flush, employing a high concentration of the contrast medium (≥370 mgI/mL), and ensuring shallow held inspiration to avoid the Valsalva maneuver [33]. Several studies have examined the additional benefits of dual-energy CT (DECT) in improving the detection accuracy and diagnostic confidence for PE [34,35]. One potential key advantage of DECT acquisition is the ability to differentiate materials based on their energy absorption characteristics; in the lungs, the parenchymal iodine distribution has been shown to correspond to lung blood volume at ventilation/perfusion scintigraphy [36]. By identifying regional iodine perfusion defects, DECT may improve the detection of peripheral pulmonary emboli [34,35]. Moreover, the DECT technique can improve the image quality even with a small amount of CA. Using monoenergetic image reconstruction, the intravascular enhancement and signal-to-noise ratio (SNR) can be optimized with significant reduction in iodine load [37].

### 3.2. Radiation Exposure in CTPA: Risks and Considerations

The primary concern regarding the use of CTPA in pregnant women is the exposure to ionizing radiation, with potential risks to the fetus depending on the stage of pregnancy and the absorbed dose. Risks are highest during the early fetal period, lower in the second trimester, and minimal in the third trimester. The periods of greatest vulnerability occur in the initial stages of pregnancy, including growth retardation between 8 and 56 days, microcephaly between 14 and 105 days, and intellectual deficits, seizures, or severe mental impairment between 56 and 105 days. Whenever possible, procedures should be postponed until the completion of major organogenesis (>12 weeks from the last menstrual period) [5]. However, advancements in CT technology have led to low radiation exposure for both mother and fetus with modern techniques. Current guidelines indicate that the estimated fetal radiation exposure during CTPA ranges from 0.05 to 0.5 milligray (mGy), while maternal radiation exposure to breast tissue is estimated to be between 3 and 10 mGy [28]. These findings align with those reported by Tromeur and colleagues in their systematic review and meta-analysis, where the absorbed fetal/uterine dose from CTPA was between 0.002 and 0.51 mGy [31]. Recently, the OPTICA (Optimized CT Pulmonary Angiography in Pregnancy) study provided new insights, estimating low mean absorbed doses: 2.9 ± 2.1 mGy for breast tissue and 0.1 ± 0.2 mGy for the uterus/fetus [38]. With current CT techniques, fetal radiation doses remain well below the threshold associated with radiation-related fetal complications, which is 50-100 mGy [39]. Furthermore, the impact on maternal cancer risk is negligible, while the lifetime cancer risk is reportedly increased by a factor of 1.0003–1.0007. Therefore, avoiding CTPA in pregnant patients with suspected PE cannot be justified solely on the basis of radiogenic cancer risks [40]. Additionally, various techniques are available to reduce radiation exposure without compromising image quality. Most of the modern CT scanners implement automatic tube current modulation (ATCM) systems and iterative reconstruction (IR) techniques, both of which have been shown to effectively reduce radiation doses [41,42]. Research on dose reduction techniques has particularly focused on lowering tube potential. The contrast between enhanced vessels and filling defects is crucial for detecting pulmonary emboli and enhancement by an iodine-based contrast medium can be augmented when tube potential is reduced. Studies suggest that using protocols at 100 peak kilovoltage (kVp) is feasible without significant loss of detail resolution, which is critical for detecting pulmonary emboli [43]. Mitchell et al. evaluated the specific contribution of contrast-monitoring techniques (test-bolus or bolus-tracked) to breast dose in both pregnant and non-pregnant women, finding that these techniques can account for 27% of the overall breast dose from a CTPA study. They also demonstrated that lowering the kVp during the contrast-monitoring phase significantly reduces breast dose without compromising CTPA quality or timing [44]. Another approach involves using a reduced z-axis technique with a limited scan range, that extends from the top of the aortic arch to just below the heart, excluding the upper and lower marginal zones [45]. A recent review on this topic concluded that this reduced scan coverage decreased radiation exposure without compromising the accuracy of PE diagnoses [46]. Furthermore, with the recent development of photon-counting-detector CT (PCD-CT) technology, the potential for radiation dose savings has further increased. Contemporary studies have explored its application for the diagnosis of acute PE [47,48]. PCD-CT offers the advantages of both spectral imaging, providing high-quality images and lung perfusion assessment, and rapid scanning with a reduced radiation exposure. Pannenbecker and colleagues demonstrated that high-pitch PCD-CT pulmonary angiography enables a significant reduction in the contrast medium (25 mL) and radiation dose (with an effective dose of 1.4 ± 0.5 mSv) for diagnosing acute PE, while maintaining good to excellent image quality compared to dual-energy pulmonary angiography on a conventional CT scanner [47].

### 3.3. Non-Contrast MRI’s Emerging Role

**Figure 1:**
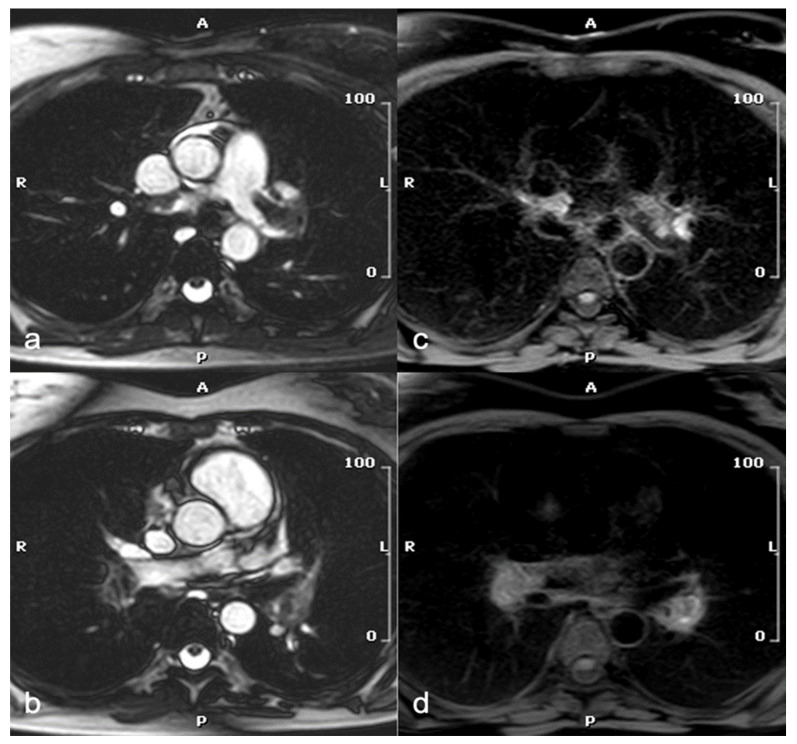
A 47-year-old woman at week 20 of pregnancy with shortness of breath and D-dimer elevation. Axial SSFP images show left and right main pulmonary artery filling defects (**a**,**b**), consistent with acute pulmonary embolism, confirmed by T1-w images with blood and fat saturation (**c**,**d**). SSFP: steady-state free precession.

Recent studies have investigated the feasibility of diagnosing PE using non-contrast MRI, an approach that would be particularly beneficial for the pregnant population, as it avoids both radiation exposure and the administration of contrast media. The balanced steady-state free precession (SSFP) sequence has proven to be of greatest interest, producing predominantly T2-weighted (T2w) contrasts, enabling the inherent distinction between embolic material and blood in patent pulmonary vessels without the need of contrast media [49] (Figure 1).

Herèdia et al. reported preliminary findings of the use of SSFP sequences for the evaluation of pregnant patients with suspected PE, demonstrating that their protocol was able to visualize central, lobar, and segmental pulmonary arteries with sufficient image quality [50]. Nyrén and colleagues developed a short, 10-min, unenhanced MRI protocol based on free-breathing, non-cardiac-gated SSFP, which demonstrated high sensitivity (90–93%) and specificity (100%), with an inter-reader agreement of 0.97 [51]. Several works have evaluated the diagnostic performance of non-contrast MRI for displaying different levels of pulmonary artery involvement in PE [52,53,54,55,56]. The results are reported in Table 1.

**Figure 2:**
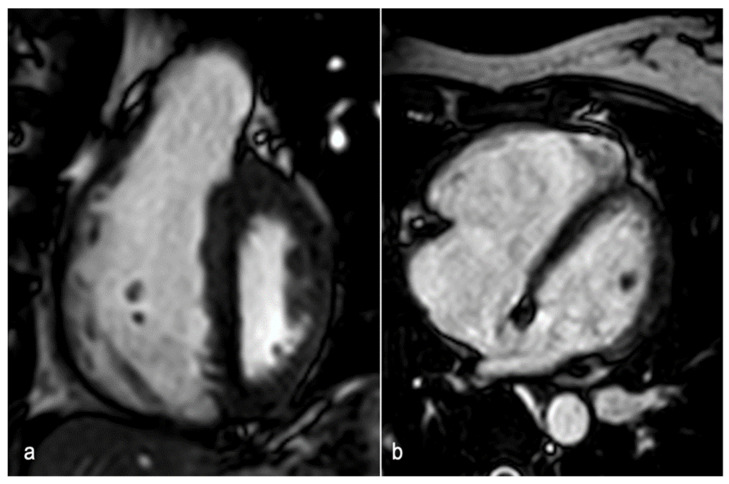
A 47-year-old woman with shortness of breath and D-dimer elevation at week 20 of pregnancy. Short-axis SSFP image (**a**) shows interventricular septum flattening in mid-systolic phase due to increased intracavitary pressure in the right ventricle. The four-chamber SSFP image (**b**) demonstrates right ventricular enlargement compared to the left ventricle. SSFP: steady-state free precession.

Additionally, the usefulness of a T1-weighted (T1w) sequence with fat and blood suppression has been investigated for visualizing embolic material. As blood clots, it goes through distinct stages that correspond to the oxygenation state of hemoglobin within red cells. During one of these stages, methemoglobin forms and acts as an endogenous contrast agent, showing a high signal on T1w sequences (Figure 1). The suppression of adipose tissue and blood signal increases contrast with the hyperintense thrombo-embolic component, facilitating clot detection. This technique shows potential for identifying fresh thrombus in cases of deep vein thrombosis and PE [57]. Furthermore, the feasibility of diffusion-weighted imaging (DWI) has been assessed for PE detection in free-breathing humans. DWI has demonstrated high sensitivity for detecting PE but is limited by poor specificity. It may serve as a useful “eye-catcher”, highlighting arteries that require closer examination. This could potentially enhance diagnostic quality, particularly at the subsegmental level, and reduce reading time [58]. MRI in the context of pulmonary embolism can also provide functional and prognostic information, with cine-SSFP sequences identifying right ventricular pressure overload, evidenced by reduced ejection fraction, increased ventricular volumes, and interventricular septal flattening (Figure 2) [59]. 

Nevertheless, MRI is not currently recommended at present for diagnosing or ruling out PE during pregnancy [28], and further research is needed to validate its clinical utility and demonstrate safety and feasibility.

## 4. Aortic Dissection (AD)

Acute AD is a rare but serious complication of pregnancy, occurring in only 0.0004% of pregnancies [60]. According to the International Registry of Acute Aortic Dissection (IRAD), pregnancy-related AD accounts for 1% of AD cases in women [61]. Although uncommon, this condition deserves attention as it may cause significant morbidity and mortality for both mother and fetus. Several heritable thoracic aortic diseases, either syndromic (such as Marfan syndrome, vascular Ehlers–Danlos syndrome, and Loeys–Dietz syndrome) or nonsyndromic, predispose individuals to both aneurysm formation and AD. Other types of congenital heart disease (e.g., bicuspid aortic valve and aortic coarctation) may also be associated with aortic dilatation, and, finally, aortic pathology may occur. Additional risk factors are hypertension and advanced maternal age [5]. Pregnancy itself is an independent risk factor for both aortic growth and AD in women with underlying aortopathy. Kamel et al. reported a fourfold increased risk of aortic dissection or rupture during pregnancy compared to during the non-pregnant state [62]. The increased risk is likely due to hormonal and hemodynamic changes in pregnancy and postpartum, including increased blood volume, cardiac output, and maternal heart rate, along with decreased arterial blood pressure and systemic vascular resistance [1]. The risk of pregnancy-related AD is highest during the third trimester and the early postpartum period, though it may persist for several weeks postpartum [61]. Distinguishing the common chest and abdominal symptoms of various causes that many women experience during pregnancy or postpartum from those associated with acute aortic syndrome (AAS) can be challenging, as not all dissections present with classic symptoms. When concerning symptoms arise, recognizing women at risk should raise the clinician’s suspicion of AD and prompt a rapid assessment and investigation. Computed tomography angiography (CTA) is the preferred imaging technique for a fast and accurate diagnosis of AD. Although MRI plays a more limited role, primarily due to its reduced availability in emergency situations, it may offer significant benefits for pregnant patients.

### 4.1. CTA: Challenges and Approaches in Clinical Practice

**Figure 3:**
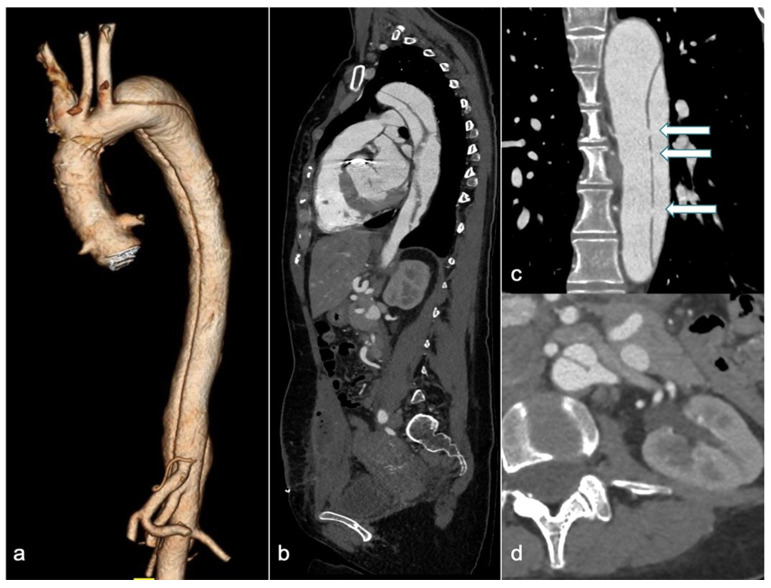
A 31-year-old woman with Marfan syndrome and a history of ascending aortic dissection, previously treated with a Bentall procedure, presented with chest pain a few days postpartum. CTA revealed an extension of the dissection into the descending thoracic and abdominal aorta (3D volume rendering in (**a**), and sagittal and coronal views in (**b** and **c**), respectively), with simultaneous enhancement of both the true and false lumens due to multiple intimal fenestrations (arrows in (**c**)). The sagittal view (**b**) shows a heterogeneous collection into the rectus abdominis muscles following a recent cesarean section. Axial image (**d**) reveals the left renal artery originating across both the true and false lumens. CTA: computed tomography angiography and 3D-VR: 3D volume rendering.

CTA is the first-line imaging modality for diagnosing suspected AD, due to its wide availability, high accuracy, short acquisition time, and ability to provide a comprehensive assessment of the thoracoabdominal aorta (Figure 3) [63].

However, in pregnant patients, the primary concern is fetal radiation exposure, especially if the fetus is included in the primary radiation field to evaluate the abdominal extent of the AD. Advances in imaging acquisition and processing over the past decade have significantly reduced radiation doses. The effectiveness of ATCM systems and IR techniques in reducing the radiation dose has been well established [64]. In recent years, studies have also demonstrated the feasibility of low tube potential protocols for aortic CTA [65,66]. Fink et al. reported that whole-aortic CTA can be performed with both very low radiation and iodine doses, while maintaining diagnostic image quality, by employing a body mass index (BMI)-adapted protocol [67]. With the introduction and implementation of dual-source CT (DSCT) and DECT technology, the potential savings in radiation and iodine dose for aortic CTA have continuously increased. The use of a DSCT high-pitch protocol for dose reduction has been reported. This protocol decreases the scan time for the entire aorta to approximately 2 s, resulting in a very low radiation dose [68]. Apfaltrer et al. demonstrated that high-pitch CTA of the aorta results in a 45–50% reduction in radiation exposure, along with contrast medium savings, while maintaining vessel attenuation at a diagnostic level [69]. DECT, by comparing two different energy levels, distinguishes materials based on their effective atomic numbers. Virtual non-contrast (VNC) images have been used in AAS imaging protocols for the diagnosis of aortic intramural hematoma (IMH) by subtracting the iodine attenuation from contrast-enhanced scans [70]. This approach demonstrates excellent diagnostic performance while reducing the number of acquisitions and achieving a mean effective dose reduction of 40% [71]. Moreover, various institutions have established protocols to minimize radiation exposure through decision-making algorithms and careful interdisciplinary collaboration. Patel and colleagues designed a specific protocol for patients with suspected AAS who had no prior history of aortic disease and no abdominal or pelvic symptoms. This protocol involved an initial CTA of the chest, which was reviewed by a monitoring radiologist while the patient remained on the scanner table. If no aortic pathology was found, the examination was terminated; conversely, if pathology was detected, a CTA of the abdomen and pelvis was immediately performed. This approach reduced radiation exposure by up to 14.6% [72]. Such strategies are particularly useful for pregnant women, as they can help avoid including the fetus in the primary radiation field whenever possible. Further research is required.

### 4.2. Unenhanced MRI: When and How

**Figure 4:**
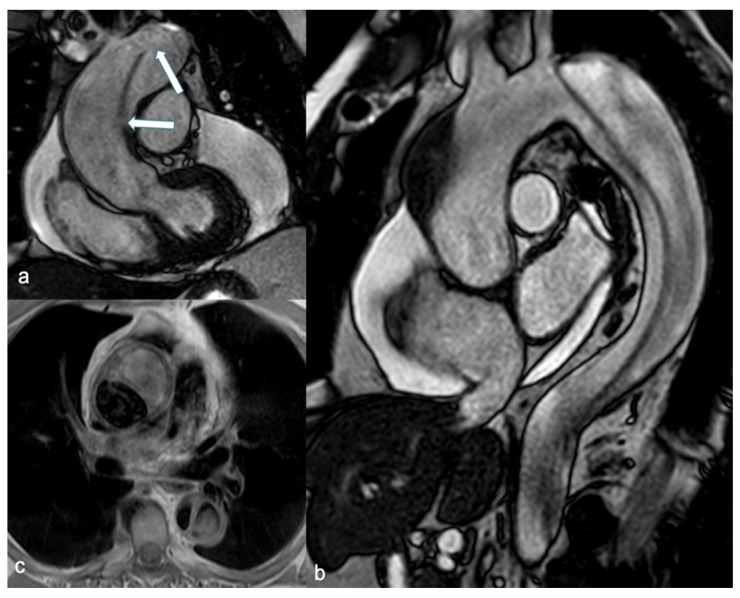
SSFP breath-hold images in coronal (**a**) and sagittal (**b**) views demonstrate an intimal-medial flap involving the entire thoracic aorta, including the ascending aorta, consistent with a Stanford Type A dissection. Multiple intimal tears are visible within the flap (arrow in (**a**)), and massive circumferential pericardial fluid is associated. An axial SE-BB image (**c**) shows a hypointense true lumen and increased signal intensity in the false lumen due to slow flow. SSFP: steady-state free precession and SE-BB: spin echo-black blood.

MRI provides comprehensive imaging of the entire aorta and its branching vessels, characterizing aortic wall changes and offering physiological evaluation of valve function, along with flow quantification. These attributes position MRI as a valuable alternative to CT. In the elective setting, the use of MRI without gadolinium is recommended in pregnant patients with aortic disease who require surveillance imaging of the aortic arch, descending aorta, abdominal aorta, or all three (Class I, Level of Evidence C) [5]. However, limitations include lower accessibility, longer examination times, reliance on patient cooperation, and challenges in monitoring and treating unstable patients during the procedure. Consequently, MRI is not commonly utilized in emergency departments (ED) for diagnosing AAS [73]. Nonetheless, it could be particularly beneficial for pregnant women with suspected AD, especially considering the availability of techniques for aortic depiction that do not require the administration of CAs. Spin echo-black blood acquisitions enable clear delineation of aortic shape and diameter, as well as the identification of wall structure alterations. The false lumen can be identified by increased intraluminal signal intensity, attributable to slow flow, and can be characterized by web-like remnants of dissected media (Figure 4) [74]. 

Unenhanced SSFP sequences provide accurate and reproducible aortic measurements as well as clear visualization of the intimal-medial flap (Figure 4) [75]. Additionally, cine SSFP MRI can delineate the entry and exit zones of the intimal-medial flap and detect aortic regurgitation by identifying flow turbulence. Pereles et al. demonstrated that a short imaging protocol combining single-shot true fast imaging with steady-state precession (FISP) and cine true FISP sequences is promising for the initial, rapid, and accurate diagnosis of aortic dissection and aneurysm in less than four minutes [76]. Finally, phase contrast sequences can provide quantitative data on flow velocity and volume in both the true and false lumen.

## 5. Spontaneous Coronary Artery Dissection (SCAD)

SCAD is a rare but potentially lethal complication of pregnancy and represents the most common etiology of pregnancy-associated acute myocardial infarction (AMI) [77]. Pregnancy-related (P-SCAD) can be categorized as antepartum, early postpartum (within 6 weeks of delivery), late postpartum (6 weeks to 12 months), and very late postpartum (12–24 months) [78]. More than 70% of cases occur during the postpartum period and the incidence is higher in multiparous women [79]. Surges in estrogen and progesterone during pregnancy, particularly with recurrent exposure in multiparity, can induce structural changes in the arterial wall, leading to weakening of the tunica media. The physiological increase in plasma volume and cardiac output during pregnancy and the increased coronary shear stresses associated with labor, may precipitate P-SCAD in vulnerable patients. Additionally, the association of breastfeeding with late and very late postpartum P-SCAD suggests that hormonal changes during lactation may compound the effects of pregnancy [78]. Beyond the pregnancy-related structural and hemodynamic changes, fibromuscular dysplasia has been strongly associated with SCAD and was documented in cases of P-SCAD [80]. The typical clinical presentation includes symptoms of acute coronary syndrome (ACS). Tweet et al. analyzed records of 323 women with SCAD, identifying 54 who were pregnant or ≤12 weeks postpartum. They compared P-SCAD with SCAD not associated with pregnancy (NP-SCAD) and found that patients with P-SCAD had a more severe clinical presentation than those with NP-SCAD, often exhibiting multivessel dissections and acute heart failure [81]. The diagnosis of SCAD is typically made by ICA in patients presenting with ACS, including pregnant women, despite concerns regarding radiation exposure [82]. Specific intracoronary imaging tools, such as optical coherence tomography (OCT) and intravascular ultrasound (IVUS), can improve and confirm diagnosis; however, some operators prefer to avoid these modalities due to the risk of propagating the dissection flap [83]. Recently, the role of coronary computed tomography angiography (CCTA) has been explored both in acute settings for low-to-intermediate risk patients and as a second-line diagnostic tool in ambiguous cases, along with non-invasive follow-up assessments [84]. The contribution of cardiac magnetic resonance (CMR) in patients with SCAD is to assess regional wall motion abnormalities and to detect the presence and extent of myocardial injury. Treatment is tailored to each individual case. In hemodynamically stable patients with limited dissections and preserved antegrade coronary flow, medical therapy has shown favorable long-term outcomes. Conversely, clinical instability, ongoing ischemia, left main stenosis, and reduced coronary flow are high-risk features that justify revascularization [85].

### 5.1. CCTA: Roles and Findings

**Figure 5:**
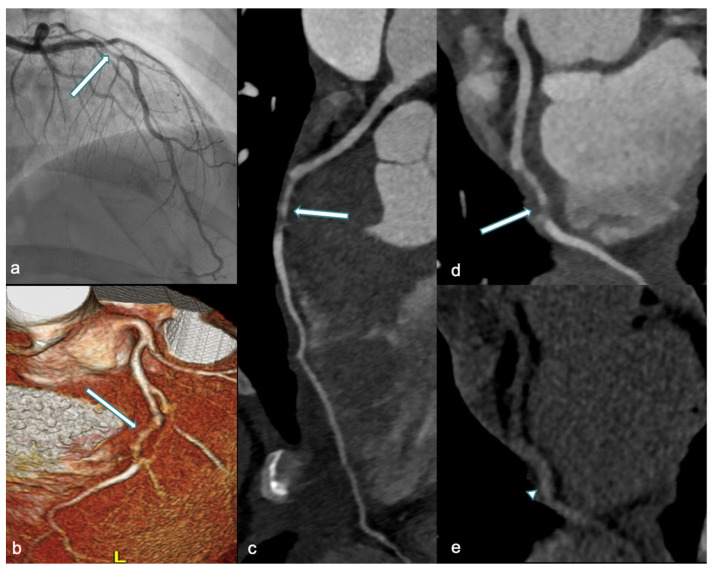
A 36-year-old woman at week 36 of pregnancy presented with chest pain and anterior ST-segment elevation on ECG. Urgent ICA (**a**) and CCTA performed one week later (3D-VR, (**b**) and curved-MPR, (**c**,**d**)) revealed a tapered luminal stenosis in the mid-LAD (white arrow), consistent with mid-LAD dissection. In (**e**), unenhanced CT shows high-density thickening of the mid-LAD wall, suggestive of IMH (arrowhead). ICA: invasive coronary angiography; CCTA: coronary computed tomography angiography; 3D-VR: three-dimensional volume rendering; MPR: multiplanar reconstruction; LAD: left anterior descending artery; and IMH: intramural hematoma.

Over the last years, the role of CCTA in assessing acute SCAD has been investigated, with several studies highlighting the utility of this imaging modality [86,87]. Pergola et al. recommend utilization of CCTA in acute settings for stable patients with a high suspicion of SCAD who do not exhibit high-risk features (i.e., ST elevation, progressive increases in serum troponin, or worsening chest pain) [84]. Moreover, the safety of CCTA makes this technique the preferred modality for follow-up in both asymptomatic and symptomatic patients. In a recent blinded study, the accuracy of CCTA in assessing coronary dissection healing was evaluated, with sensitivity and specificity found to be 72% and 53.8%, respectively. Additionally, the authors estimated that the optimal timing for CCTA to assess dissection healing was 80 days [88]. Roura and colleagues explored the feasibility of non-invasive follow-up in 34 SCAD patients, concluding that CCTA is an excellent tool that allows assessment and confirmation of vessel wall healing in most patients, particularly those who did not undergo percutaneous coronary intervention (PCI) [89]. The CCTA appearance of SCAD includes both primary and secondary findings (Figure 5), as reported in Table 2 [90].

The most common artery involved is the left anterior descending (LAD), followed by the left circumflex artery (LCX) and the right coronary artery (RCA), with multivessel disease occurring in 10%–15% of cases [91]. Despite its benefits, CCTA has certain limitations. Its accuracy is reduced for small vessels, as distal coronary arteries or side branches may be missed due to limitations in spatial and temporal resolution. Another pitfall involves motion artifacts, which can blur the vessel and the adjacent perivascular fat, mimicking a dissection flap or stenosis and potentially leading to misinterpretation as SCAD [85].

### 5.2. CMR Assessment

**Figure 6:**
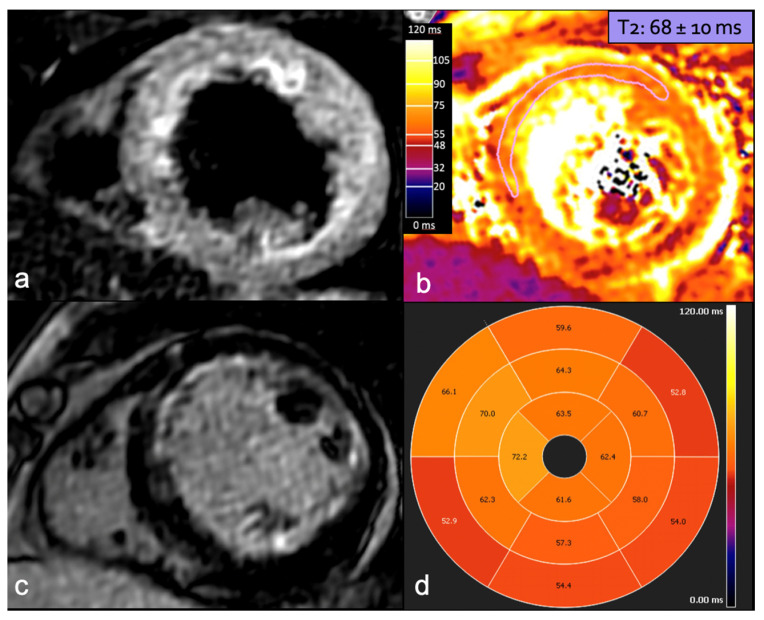
A 36-year-old woman at week 36 of pregnancy presented with chest pain and anterior ST-segment elevation on ECG. CMR detected edema in the LAD territory in STIR sequence (**a**), confirmed by increased T2 mapping values in the same territory, with maximum values in the mid-ventricular anterior and anteroseptal segments (**b**,**d**), consistent with recent infarction-related changes. LGE with ischemic subendocardial pattern in LAD territory is depicted in delayed-enhancement sequence (**c**). CMR: cardiac magnetic resonance; LAD: left anterior descending artery; STIR: short tau inversion recovery; and LGE: late gadolinium enhancement.

Emerging evidence suggests the potential diagnostic and prognostic value of CMR in managing patients with SCAD. CMR should be considered to confirm myocardial infarction when the diagnosis is uncertain, to assess the extent of myocardial involvement, and to investigate other possible etiologies and complications [92]. In a case-control study involving 158 SCAD survivors with angiographically confirmed diagnoses, Al-Hussaini and colleagues found that most patients with SCAD had no or small infarctions, with a preserved ejection fraction on follow-up CMR. However, those presenting with STEMI, reduced flow on ICA, multivessel SCAD, or signs of connective tissue disorders were more likely to have larger infarctions [93]. CMR abnormalities in SCAD patients may resemble those observed in patients with myocardial infarction from other causes. A standard CMR protocol should include sequences that provide an adequate morpho-functional and tissue characterization [94]. Cine-MRI sequences (two-dimensional SSFP technique) enable the assessment of wall thickness, ventricular volumes, ejection fraction (EF), and wall motion abnormalities in the affected coronary territory [90]. The presence of myocardial edema can be detected in T2-weighted short tau inversion recovery (STIR T2w) images as wall hyperintensity. Perfusion defects in resting perfusion study and microvascular obstruction in early gadolinium enhancement (EGE) imaging may be identified. The evaluation of myocardial viability is conducted using delayed-enhancement sequences, which can reveal areas of late gadolinium enhancement (LGE) with subendocardial or transmural patterns in the involved coronary territory (Figure 6) [95].

Finally, the implementation of quantitative T1- and T2-mapping techniques has significantly enhanced the accuracy of detecting myocardial edema and scar tissue, providing valuable insights into the extent of cardiac damage and aiding in the assessment of myocardial viability.

## 6. Peripartum Cardiomyopathy (PPCM)

PPCM is defined as an idiopathic cardiomyopathy occurring towards the end of pregnancy or in the months following delivery, abortion, or miscarriage, characterized by a left ventricular ejection fraction (LVEF) of less than 45% in the absence of other causes of heart failure [96]. Although it is the most common cardiomyopathy in pregnancy [97], PPCM remains a relatively rare condition with an incidence in the U.S. ranging from 1 in 1000 to 1 in 4000, possibly rising due to factors like increasing maternal age, multifetal pregnancies, and better disease recognition [98]. Other risk factors are African ancestry, pre-eclampsia, hypertension, subsequent pregnancy after PPCM, a family history of cardiomyopathy, smoking, diabetes, malnutrition, and prolonged use of tocolytic beta-agonists [99]. The etiopathogenesis remains unknown, though proposed mechanisms include genetic predispositions, myocarditis, abnormal immune responses to pregnancy, pathological responses to the hemodynamic changes in pregnancy, hormonal imbalances, angiogenic dysregulation, stress-activated cytokines, and nutritional deficiencies [100]. PPCM commonly presents with signs and symptoms of heart failure (HF) that can be mistaken for normal pregnancy-related changes, potentially delaying diagnosis. Persistence or disproportionate severity of symptoms should always be considered suspicious and promptly investigated, as delays increase the risk of complications and worsen outcomes. Most women are diagnosed postpartum, typically within the first month after delivery, whereas a small subset present during the second and third trimesters of pregnancy [101]. No specific biomarkers for PPCM are currently available [99], but B-type natriuretic peptide (BNP) and N-terminal pro-BNP levels can help rule out heart failure, as they remain low in normal pregnancy, whereas they are consistently elevated in PPCM patients [102]. PPCM remains a diagnosis of exclusion in women presenting with HF signs and symptoms and unexplained systolic dysfunction [100]. Cardiac imaging plays a fundamental role in diagnosing HF in peripartum women, nevertheless aiding in the exclusion of life-threatening complications, guiding treatment, and providing prognostic information. Transthoracic echocardiography (TTE) is the key diagnostic tool for confirming or excluding PPCM and should be performed in every pregnant woman with HF symptoms, because of its availability and safety [103]. However, factors such as the heart’s horizontal position caused by increased abdominal pressure during pregnancy, along with obesity, which is common in PPCM patients, may compromise the TTE images’ quality. CMR can overcome these limitations and plays a growing role in managing these patients, providing detailed tissue characterization.

### CMR: A Key to Diagnostic and Prognostic Insights

**Figure 7:**
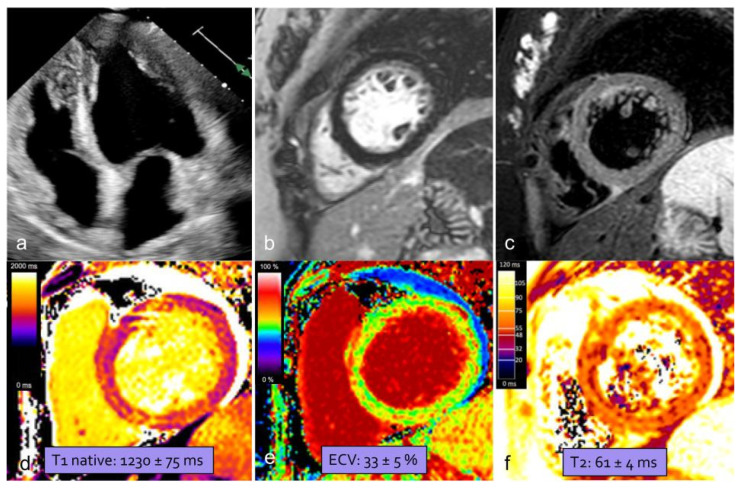
A 38-year-old woman presented with chest oppression and hypertension eight days after delivery. TTE (**a**) showed decreased LVEF (45%) and global left ventricle hypokinesia. CMR confirmed left ventricle disfunction, but no focal delayed enhancement was identified on LGE imaging (**b**) and STIR images (**c**) showed faint and diffuse hyperintensity. Quantitative mapping showed high global myocardial native T1, ECV, and T2 values ((**d**–**f**): mean value of the basal segments shown in the Figures). TTE: transthoracic echocardiogram; LVEF: left ventricular ejection fraction; CMR: cardiac magnetic resonance; LGE: late gadolinium enhancement; STIR: short tau inversion recovery; and ECV: extracellular volume.

According to the Society of Cardiovascular Magnetic Imaging (SCMR), CMR is the recommended method for diagnosing and assessing the severity of PPCM, as it provides valuable information about myocardial damage, which is essential for predicting cardiac dysfunction and stratifying risk in both current and future pregnancies [94]. CMR imaging is the gold standard for non-invasive measurement of left ventricle (LV) and right ventricle (RV) volumes and ejection fractions, offering operator-independent assessments without ionizing radiation [104]. In PPCM, CMR can reveal LV impairments (LVEF<45%) and a dilated cardiac phenotype, characterized by increased volumes in both the left and right chambers. A recent meta-analysis, published by Prameswari et al., reported that the average LV end-diastolic volume index (EDVi) in PPCM patients was approximately 122.5 mL/m^2^ [105], significantly higher than normal values assessed in healthy pregnant women in the third trimester and postpartum period [106]. Moreover, since evaluation of RV parameters by echocardiography may be inaccurate, further evaluation by CMR is recommended for a comprehensive and more precise assessment of right ventricular parameters [107]. RV dysfunction (RV < 40%) is a negative predictor of disease outcomes: patients with impaired RV function tend to have a less favorable prognosis for cardiac recovery as it is associated with persistent LV systolic impairment and poor outcomes [108]. An additional advantage of CMR is its ability to provide detailed tissue characterization and accurate quantification of myocardial injury (Figure 7). 

The prevalence of myocardial edema—detected by high signal intensity on T2w images and increased T1 and T2 mapping values—varies in the literature. It is more common in the acute phase of PPCM and may indicate a potentially reversible myocardial injury. Persistent myocardial edema is associated with an increased risk of developing poor left ventricular function recovery [105]. To the best of our knowledge, only two studies have analyzed native T1 mapping, T2 mapping, and extracellular volume (ECV) values in PPCM, with partially consistent findings. Liang et al. found an increase in all three values in PPCM patients compared to healthy controls. They also found that baseline values were higher in unrecovered patients than in recovered ones, suggesting a potential prognostic role for these markers in PPCM [109]. LGE detection is fundamental to assess prognosis and functional recovery in PPCM patients as its presence is associated with a poor prognosis, correlating with worsening ventricular function over time or lack of functional recovery [105,107,110]. The prevalence of LGE in PPCM patients varies widely across studies, ranging from 5% [111] to 71% [107], with variability likely due to differences in cohort size, racial and genetic factors, and timing of assessments. Supporting the hypothesis of the time dependence of detection of tissue composition alteration, LGE was more likely to be present in follow-up scans in comparison to acute-phase scans [105]. When identified, LGE typically exhibits a non-ischemic pattern, with a linear or patchy distribution, primarily localizing in subepicardial and intramural areas [112]. The localization of LGE in the left ventricle is also variable, with a prevalence in the basal- and mid-ventricular antero-septal regions [107,112]. Finally, CMR is useful in the detection of intracardiac thrombi, a relatively common complication in PPCM [113]. The suggested CMR protocol for patients with PPCM is shown in Table 3, with the administration of GBCAs reserved for the postpartum period [94].

## 7. Conclusions

Cardiovascular imaging techniques, particularly CT and MRI, serve as fundamental tools in the assessment and diagnosis of pregnancy-related CVDs, providing critical insights that inform clinical decisions and guide appropriate management strategies. However, the management of these conditions presents unique challenges that require a careful balance between accurate diagnosis and the safety of the mother and the fetus. This review aims to increase the awareness of radiologists and clinicians on pregnancy-related CVDs and the potential risks associated with radiation exposure and the administration of CAs during gestation and lactation in order to optimize imaging techniques’ selection, ensuring precise diagnosis while safeguarding the health of both the mother and fetus.

## Figures and Tables

**Table 1:** Overview of studies on non-contrast MRI diagnostic performance in pulmonary embolism [52,53,54,55,56].

Study	Sensitivity	Specificity	PPV	NPV
Kalb et al., 2012 [52]	67%	100%	100%	83%
Pasin et al., 2017 [53]	85.0%	98.6%	94.5%	95.9%
Fu et al., 2021 [54]	81.3%	80%	92.9%	57.1%
Mohammad et al., 2023 [55]	84%	100%	100%	79.2%
Medson et al., 2024 [56]	97.87%	100%	100%	99.49%

PPV: positive predictive value and NPV: negative predictive value.

**Table 2:** CCTA Findings of SCAD [90].

Primary Findings			CCTA appearance
	Abrupt luminal stenosis	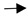	>50% diameter change over a length of 0.5 mm
	Tapered luminal stenosis	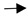	>50% diameter change over a length of 5.0 mm
	IMH	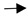	Vessel wall thickening (hyperattenuation in unenhanced CT)
	Dissection	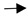	Linear hypoattenuation extending between contrast medium–filled false and true lumens
Secondary Findings	Epicardial and perivascular fat stranding Coronary tortuosity Myocardial hypoperfusion Absence of coronary calcifications Vessel occlusion with no distal flow present		

CCTA: coronary computed tomography angiography and IMH: intramural hematoma.

**Table 3:** Suggested CMR protocol for PPCM patients.

CMR Sequences			Planes	Evaluation/Detection
**Cine-SSFP**			SA and long axis	Cardiac structure and function
**T2w**		T2w imaging	SA	Myocardial edema
		T2 mapping	SA	Myocardial edema
**T1w**	Pre-contrast	Native T1 mapping	SA	Myocardial edema, necrosis and fibrosis
	Post-contrast (postpartum period)	EGE	SA	Hyperemia/capillary leak and thrombus
		LGE	SA and long axis	Myocardial edema, necrosis, fibrosis and thrombus
		Enhanced T1 mapping/ECV	SA	Myocardial edema, necrosis and fibrosis

Cine-SSFP: steady-state free precession; SA: short axis plane; EGE: early gadolinium enhancement; ECV: extracellular volume; and LGE: late gadolinium enhancement.

## Data Availability

No new data were created or analyzed in this study.
